# Supplementary material for: Mutant p53 regulates a distinct gene set by a mode of genome occupancy that is shared with wild type
Source: EMBO Rep. 2025 Jan 28;26(5):1315–43. doi: 10.1038/s44319-025-00375-y (PMC11893899; doi:10.1038/s44319-025-00375-y)
Supplement: Supplementary file 1 — Appendix [file 44319_2025_375_MOESM1_ESM.pdf]

Appendix *for*

Wild type and tumor-derived mutant p53 share a non-canonical mode of gene occupancy

Table of Contents

Page

2 Appendix Figure 1. p53 cannot be detected by immunoblotting in bone marrows.

3 Appendix Figure 2. Mutant p53 and Mdm2 only inhibit a subset of downregulated wild-type p53 gene expression.

4 Appendix Figure 3. Truncated p53 can be detected by immunoblotting in Δ10/Δ10 or Δ19/Δ19 mice.

5 Appendix Figure 4. The binding site motifs for Nuclear Respiratory Factor 1 (encoded by the mouse Nrf1 gene) are distinct from those of Nuclear Factor, Erythroid 2-Like 1 (encoded by the mouse *Nfe2l1* gene).

6 Appendix Figure 5. In the absence of radiation treatment, mutant p53-expressing bone marrow shows distinct gene expression when compared to that of the p53-null.

7 Appendix Figure 6. Gene expression after 9.5Gy X-radiation is p53-dependent in bone marrows of 172H/+ mice.

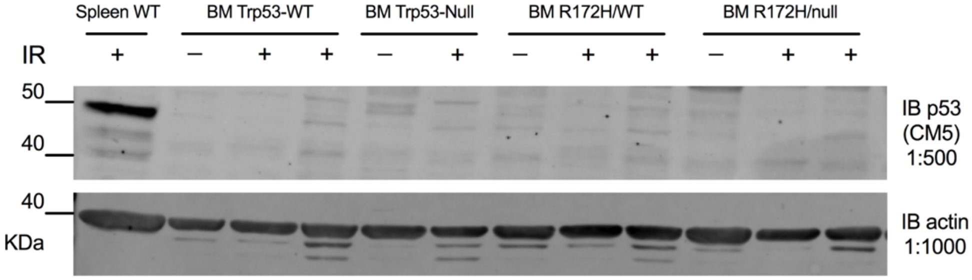

Appendix Figure 1. p53 cannot be detected by immunoblotting in bone marrows.
8-week old mice of the indicated genotype were untreated to treated with 9.5Gy of X-ray. After 3h, protein was extracted from bone marrow, and subjected to immunoblotting. Each lane represents tissue from a single animal.

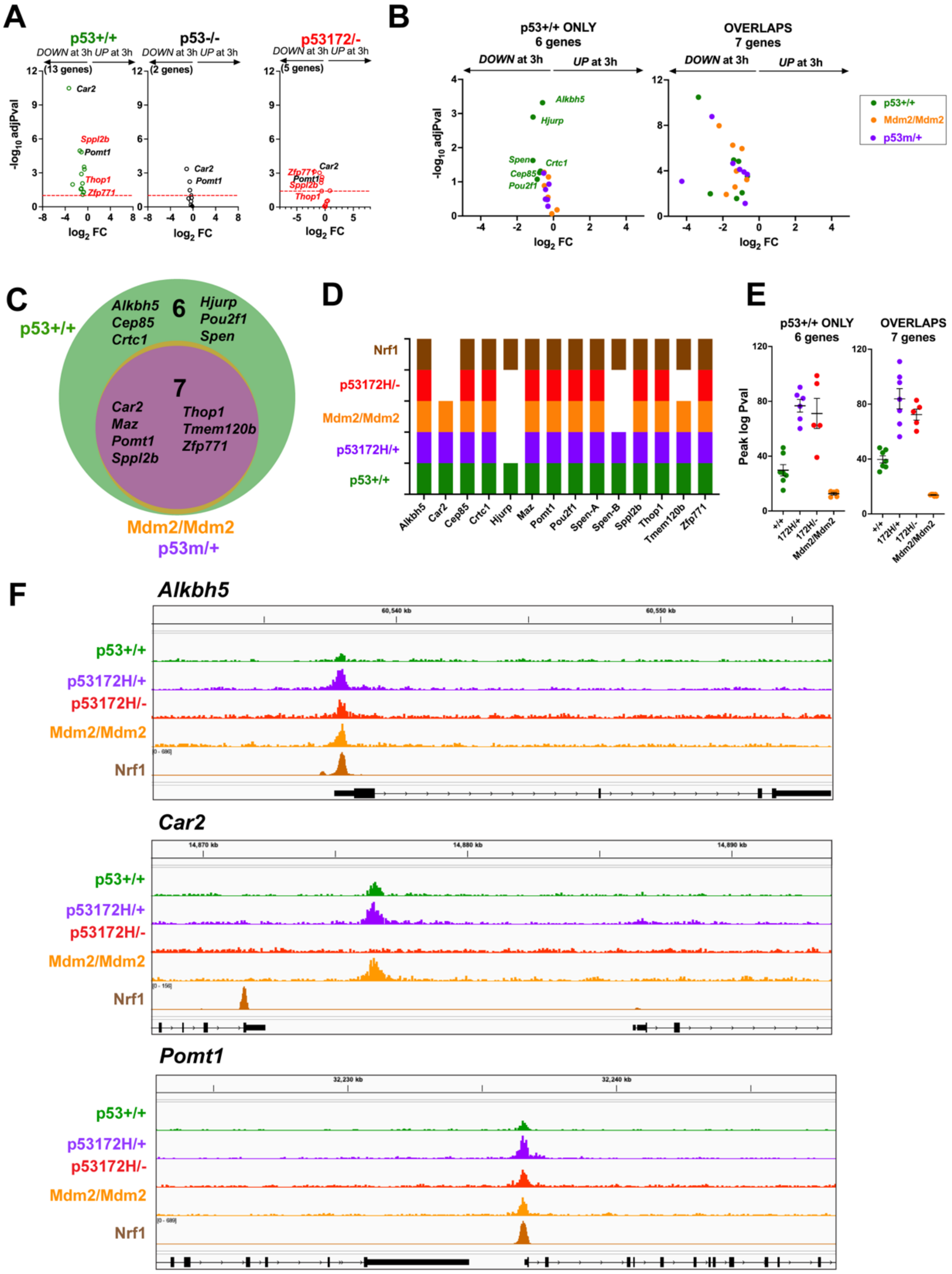

Appendix Figure 2. Mutant p53 and Mdm2 only inhibit a subset of downregulated wild-type p53 gene expression.
(A) The expression of the 13 downregulated genes in p53+/+ bone marrow was examined in either p53-/- or p53172H/- mice. Corresponding Volcano plots are shown
(B) The expression of the 13 downregulated genes in p53+/+ bone marrow was examined in either p53172H/+ or the Mdm2/Mdm2 mice. Corresponding Volcano plats are shown. Two classes of genes are identified: those which are only downregulated in p53+/+ mice (left) and those which also differentially regulated in either the p53172H/+ or the Mdm2/Mdm2 mice (right).
(C) A Venn diagram shows the two classes of genes identified in (B)
(D) For each of the 13 downregulated genes in p53+/+ bone marrow, the presence of a corresponding ChIP peak for each indicated genotype is shown. Nrf1 occupancy is taken from an ENCODE data set for MEL cells (Accession: ENCSR135SWH).
(E) A plot of the corresponding adjPval for the ChIP peaks associated with each gene set from the ChIP-seq analysis for each genotype is shown.
(F) ChIP profiles of three such genes visualized in the IGV Browser are shown.

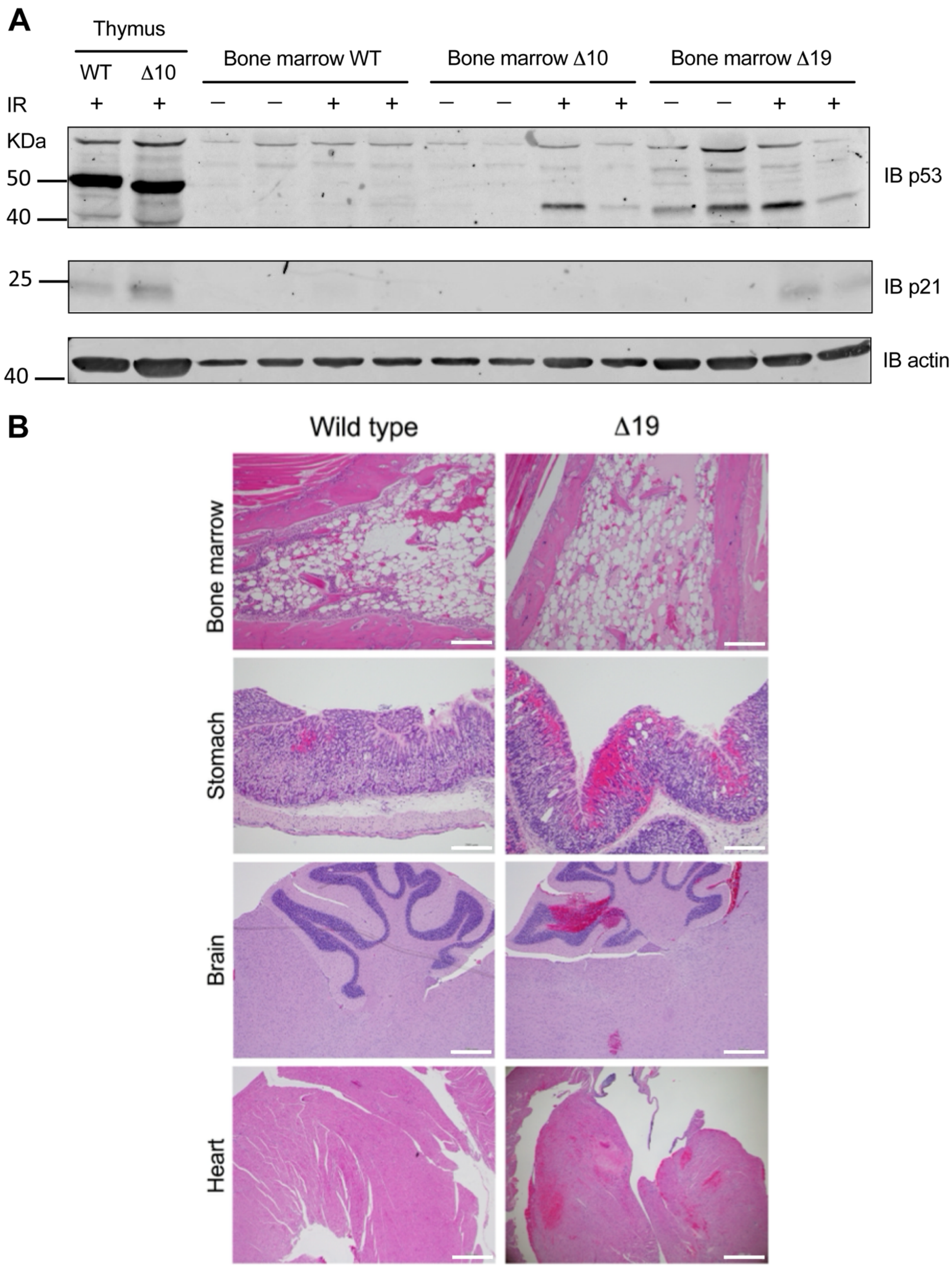

Appendix Figure 3. Truncated p53 can be detected by immunoblotting in Δ10/Δ10 or Δ19/Δ19 mice.
(A) 8-week old mice of the indicated genotype were untreated to treated with 6Gy of X-ray. After 3h, protein was extracted from bone marrow, and subjected to immunoblotting. Each lane represents tissue from a single animal.
(B) 8-week old mice of the indicated genotype were untreated to treated with 6Gy of X-ray. H&E sections of the indicated tissues are shown. Scale bars show 100µm.

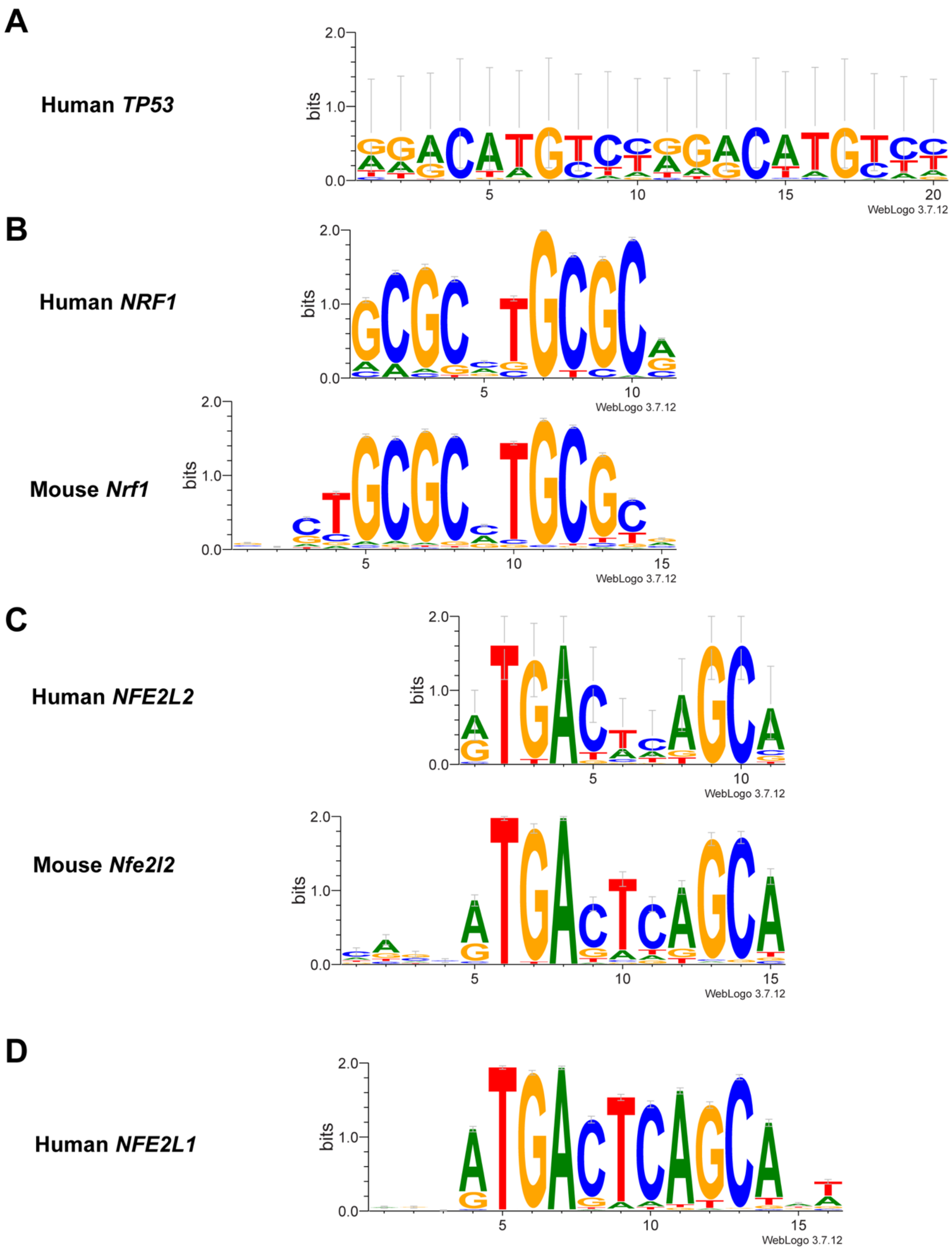

Appendix Figure 4. The binding site motifs for Nuclear Respiratory Factor 1 (encoded by the mouse Nrf1 gene) are distinct from those of Nuclear Factor, Erythroid 2-Like 1 (encoded by the mouse *Nfe2l1* gene).
(A) The M01651 matrix (TRANSFAC Database) for human p53 is shown.
(B) The MA0506.1 and MA0506.2 matrices (JASPAR Database) for Nuclear Respiratory Factor 1 encoded by the human *NRF1* gene and the mouse *Nrf1* gene, respectively are shown.
(C) The matrices MA0150.1 and MA0150.2 (JASPAR Database) for Nuclear Factor, Erythroid 2-Like 2, encoded by the human *NFE2L2* gene and the mouse *Nfe2l2* gene, respectively are shown.
(D) The MA0089.2 matrix (JASPAR Database) for Nuclear Factor, Erythroid 2-Like 1, encoded by the human *NFE2L1* gene is shown.

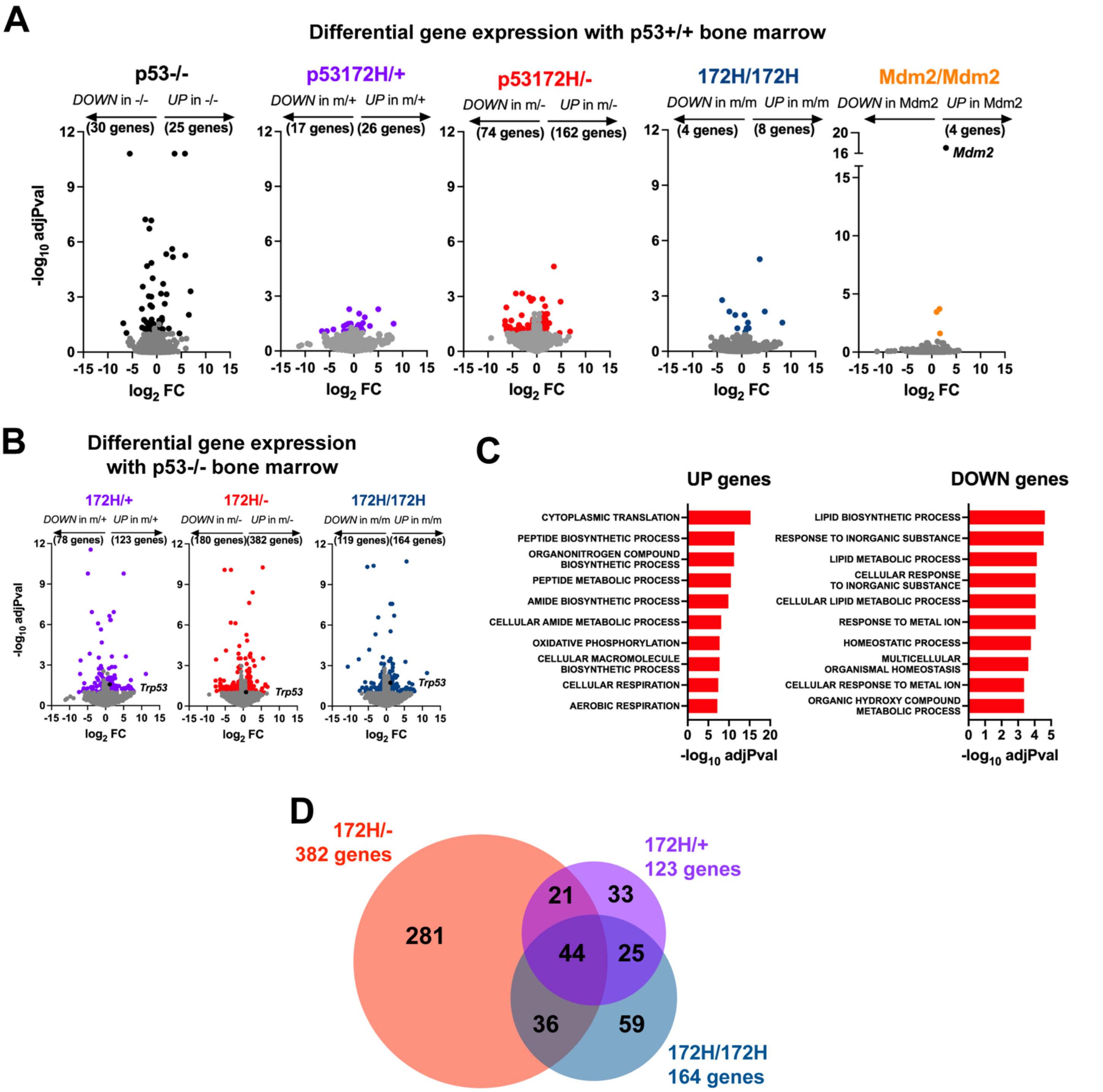

Appendix Figure 5. In the absence of radiation treatment, mutant p53-expressing bone marrow shows distinct gene expression when compared to that of the p53-null.

(A) Differential gene expression comparing untreated wild-type mice to each of the indicated genotypes is shown as Volcano plots

(B) Differential gene expression comparing untreated p53-null mice to each of the indicated genotypes is shown as Volcano plots.

(C) GSEA on the 382 up or 180 down genes in p53m/+ mice compared to the p53-null gave indicated GO:BP terms.

(D) A Venn diagram is shown for the genes that are upregulated in the indicated genotypes (shown in B) when compared to p53 null mice.

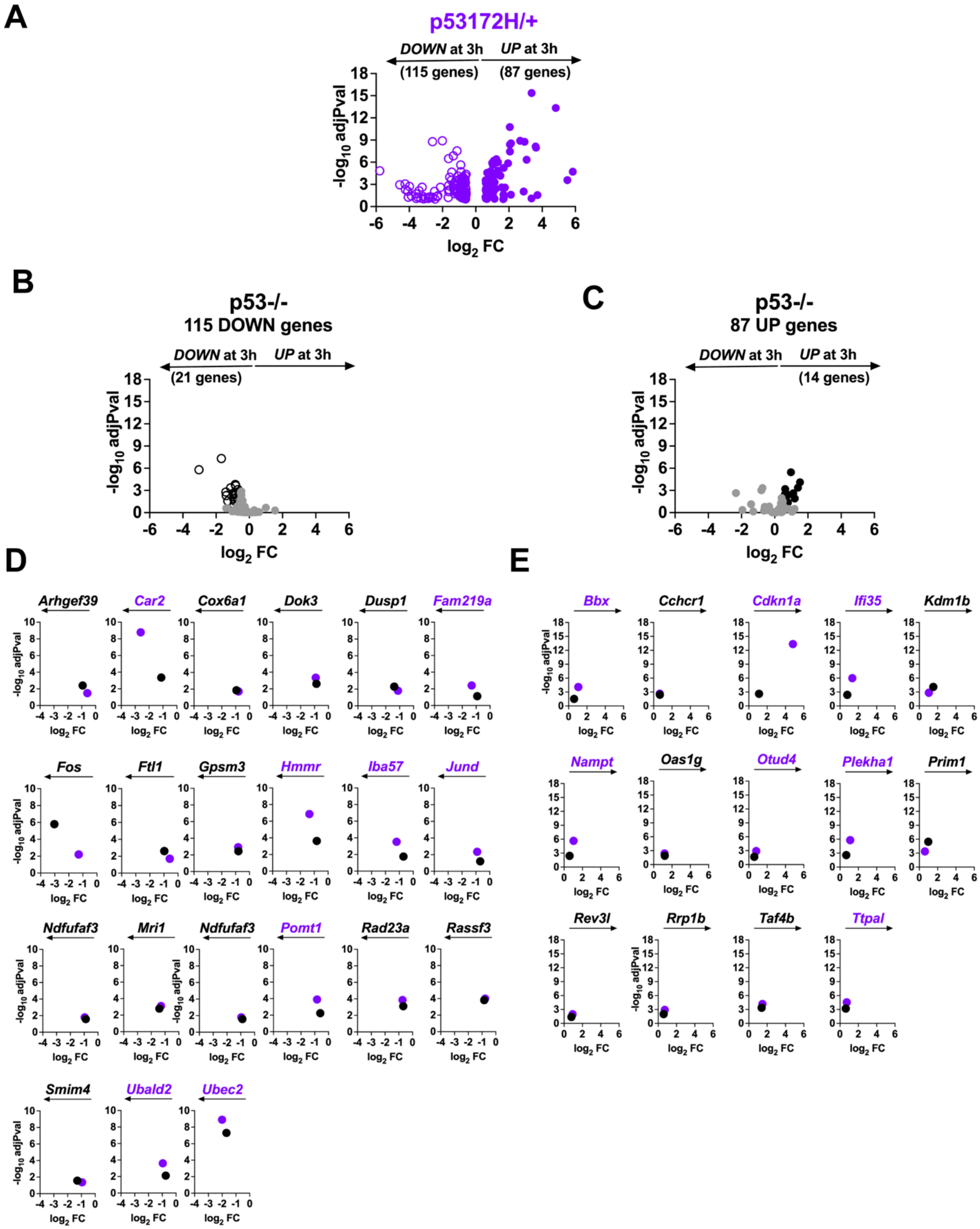

Appendix Figure 6. Gene expression after 9.5Gy X-radiation is p53-dependent in bone marrows of 172H/+ mice.
(A) 87 genes are up and bound, and 115 genes are down and bound at 3h after 9.5Gy in bone marrows of 172H/+ mice. Data is derived from N=3 mice. Genes with an adjPval <.1 are in color.
(B) For the 115 down-regulated genes, a Volcano plot for p53-/- mice is shown.
(C) For the 87 upregulated genes, Volcano plots for p53-/- mice is shown.
(D) For the 21 significantly down-regulated genes in (B), individual Volcano plots showing p53-/- mice (black) or p53m/+ mice (purple) are shown. 13 of these show differential expression in p53 null mice. The gene names of these are in purple. Thus, of the 115 downregulated genes in (A), only 102 are p53-dependent.
(D) For the 14 significantly up-regulated genes in (C), individual Volcano plots showing p53-/- mice (black) or p53m/+ mice (purple) are shown. 7 of these show differential expression in p53 null mice. The gene names of these are in purple. Thus, of the 87 upregulated genes in (A), only 80 are p53-dependent.
