# Supplementary material for: Mutant p53 regulates a distinct gene set by a mode of genome occupancy that is shared with wild type
Source: EMBO Rep. 2025 Jan 28;26(5):1315–43. doi: 10.1038/s44319-025-00375-y (PMC11893899; doi:10.1038/s44319-025-00375-y)
Supplement: Supplementary file 10 — Expanded View Figures [file 44319_2025_375_MOESM10_ESM.pdf]

Expanded View Figures

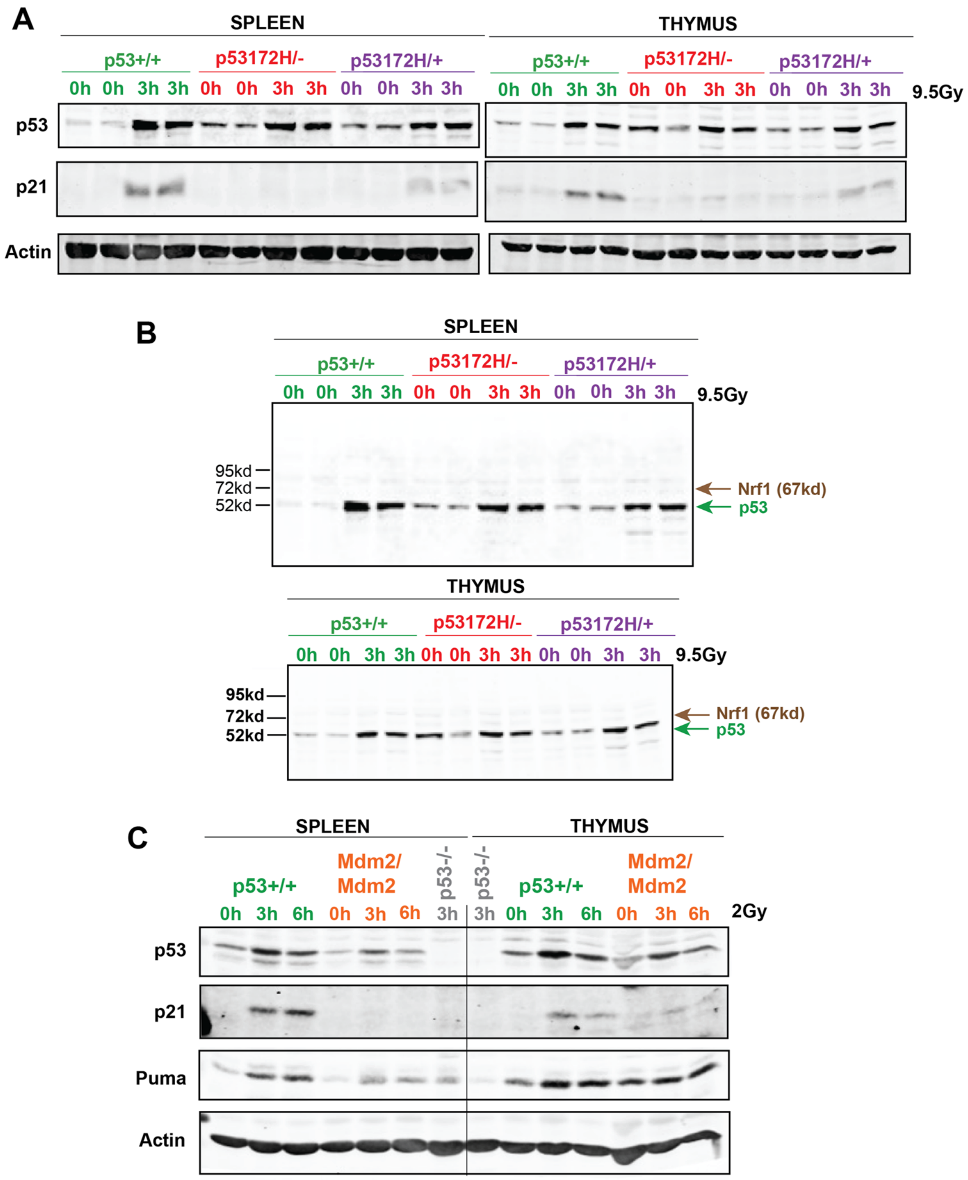

Figure EV1. p53 is detectable in spleen and thymus of mutant p53 and Mdm2-overexpressing mice.

8-week old mice of the indicated genotype were untreated or treated with shown doses of X-ray. After the indicated time points, protein was extracted from spleen and thymus, and subjected to immunoblotting. Each lane represents tissue from a single animal. (**A**) Mutant p53 protein is induced by X-radiation in mouse spleen and thymus. Extracts from wild-type (p53+/+), heterozygous mutant (p53172H/+) or hemizygous mutant (p53172H/−) spleen and thymus were immunoblotted with antibodies against either p53, p21, or Actin as indicated. (**B**) The antibody to p53 does not detect a band with the molecular weight of Nrf1 in mouse thymus or spleen. The immunoblots in (**A**) that were probed with the antibody to p53 prior to cropping are shown. The size of mouse Nrf1 (67kd) is indicated on the right. The position of standard molecular weight markers is shown on the left. (**C**) p53, p21, and Puma protein are induced by X-radiation in Mdm2 transgenic spleen and thymus. Extracts from wild-type (p53+/+), transgenic Mdm2 (Mdm2/Mdm2), or p53 null (p53−/−) spleen and thymus were immunoblotted with antibodies against either p53, p21, Puma, or Actin as indicated.

 © The Author(s)

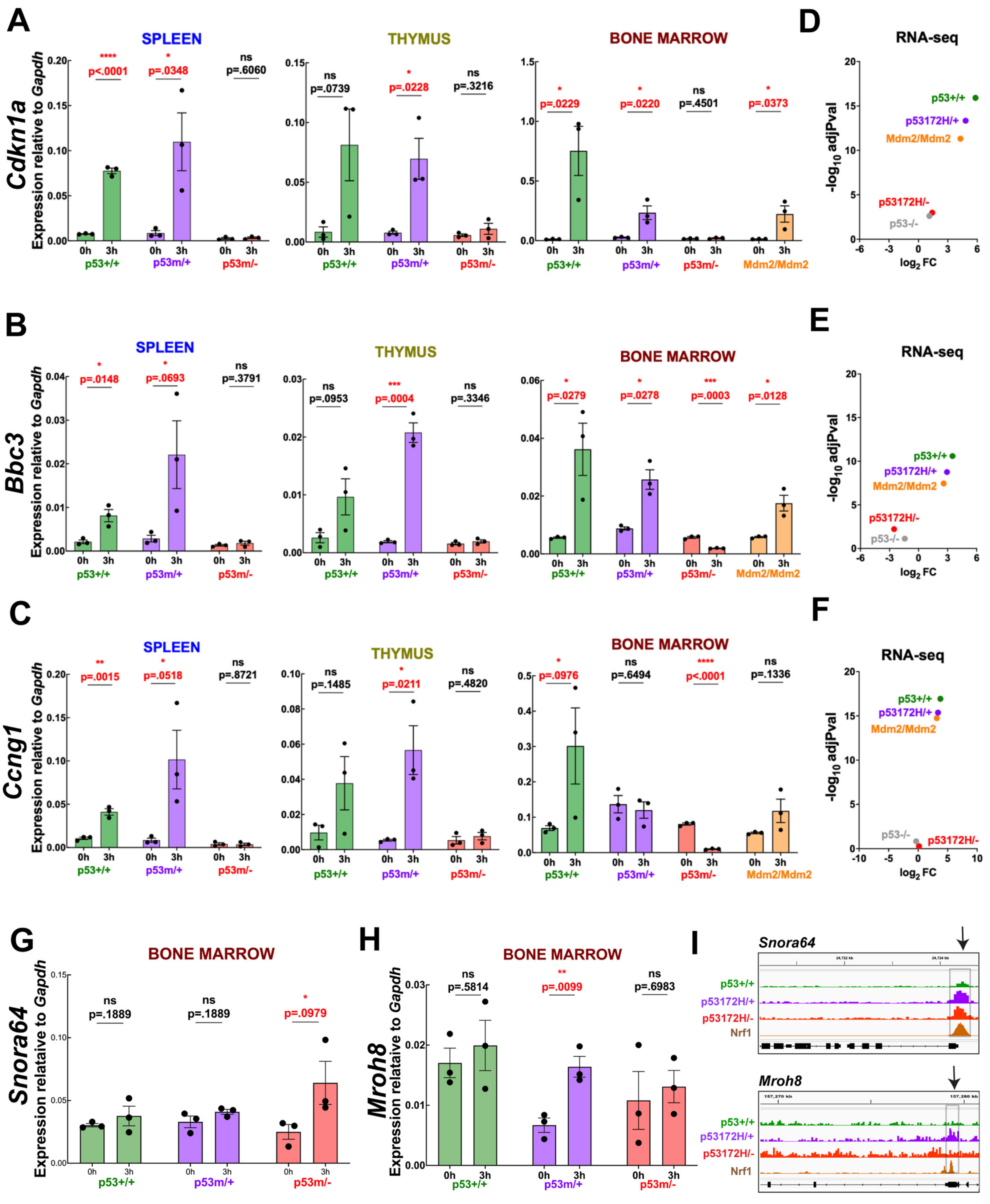
A
SPLEEN
THYMUS
BONE MARROW
Cdkn1a
Expression relative to Gapdh
p<.0001
p=.0348
p=.6060
p=.0739
p=.0228
p=.3216
p=.0229
p=.0220
p=.4501
p=.0373
p53+/+
p53m/+
p53m/-
Mdm2/Mdm2
D
RNA-seq
-log10 adjPval
log2 FC
p53172H/+
p53172H/-
p53-/-
B
Bbc3
p=.0148
p=.0693
p=.3791
p=.0953
p=.0004
p=.3346
p=.0279
p=.0278
p=.0003
p=.0128
E
C
Ccng1
p=.0015
p=.0518
p=.8721
p=.1485
p=.0211
p=.4820
p=.0976
p=.6494
p<.0001
p=.1336
F
G
Snora64
Expression relataive to Gapdh
p=.1889
p=.1889
p=.0979
H
Mroh8
p=.5814
p=.0099
p=.6983
I
Nrf1

Figure EV2. RT-qPCR confirms gene expression changes observed by RNA-seq.

(**A–C**) 8 week old mice of the indicated genotype were untreated or treated with 9.5 Gy of X-ray. After 3 h, RNA was extracted from spleen, thymus, and bone marrow, and subjected to RT-qPCR. Expression of *Cdkn1a* (**A**), *Bbc3* (**B**), and *Ccng1* (**C**) were determined relative to *Gapdh*. Each circle represents a single biological replicate from individual mice. $N = 3$ mice for each sample. Unpaired *t* test was performed and resulting *P* values are as shown. ****$P < 0.0001$; ***$P < 0.001$; **$P < 0.01$; *$P < 0.1$; ns, not significant. Significant *P* values are shown in red. Error bars are SEM. *Cdkn1* 3 h vs 0 h. Spleen: p53 +/+, $P < 0.0001$****; p53m/+, $P = 0.0348$*; p53m/−, $P = 0.6060$(ns). Thymus: p53 +/+, $P = 0.0739$(ns); p53m/+, $P = 0.0228$*; p53m/−, $P = 0.3216$(ns). Bone Marrow: p53 +/+, $P = .0229$*; p53m/+, $P = 0.0220$*; p53m/−, $P = 0.4501$(ns); Mdm2/Mdm2, $P = 0.0373$*. *Bbc3* 3 h vs 0 h. Spleen: p53 +/+, $P = 0.0148$*; p53m/+, $P = 0.0693$*; p53m/−, $P = 0.3791$(ns). Thymus: p53 +/+, $P = 0.0953$(ns); p53m/+, $P = 0.0004$***; p53m/−, $P = 0.3346$(ns). Bone Marrow: p53 +/+, $P = 0.0279$*; p53m/+, $P = 0.0278$*; p53m/−, $P = 0.0003$***; Mdm2/Mdm2, $P = 0.0128$*. *Ccng1* 3 h vs 0 h. Spleen: p53 +/+, $P = 0.0015$**; p53m/+, $P = 0.0518$*; p53m/−, $P = 0.8721$(ns). Thymus: p53 +/+, $P = 0.1485$(ns); p53m/+, $P = 0.0211$*; p53m/−, $P = 0.4820$(ns). Bone Marrow: p53 +/+, $P = 0.0976$*; p53m/+, $P = 0.6494$(ns); p53m/−, $P < 0.0001$****; Mdm2/Mdm2, $P = 1336$(ns). (**D–F**) The corresponding RNA-seq results for *Cdkn1a* (**D**), *Bbc3* (**E**), and *Ccng1* (**F**) in bone marrow are shown. Statistical analysis for differential gene expression analysis was performed using DESeq2 (Love et al, 2014). (**G**, **H**) 8 week old mice of the indicated genotype were untreated or treated with 9.5 Gy of X-ray. After 3 h, RNA was extracted from bone marrow, and subjected to RT-qPCR. Expression of S*nora64* (**G**) and *Mroh8* (**H**) were determined relative to *Gapdh*. Each circle represents a single biological replicate from individual mice. Unpaired *t* test was performed and resulting *P* values are as shown. ****$P < 0.0001$; ***$P < 0.001$; **$P < 0.01$; *$P < 0.1$; ns, not significant. Significant *P* values are shown in red. Error bars are SEM. *Snora64* 3 h vs 0 h: p53 +/+, p-0.1889(ns); p53m/+, $P = 0.1889$(ns); p53m/−, $P = 0.0979$*. *Mroh8* 3 h vs 0 h: p53 +/+, $P = 0.5814$(ns); p53m/+, $P = 0.0099$**; p53m/−, $P = 0.6983$(ns). (**I**) 8 week-old mice of the indicated genotype were treated with 9.5 Gy of X-ray. After 3 h, bone marrows were subjected to cross-linking and ChIP-seq analyses. ChIP profiles of S*nora64* and *Mroh8* visualized in the IGV Browser are shown. Nrf1 occupancy is taken from an ENCODE data set for MEL cells (Accession: ENCSR135SWH). Data information: In (**A–C**, **G**, **H**), data are presented as mean ± SEM. *$P < 0.1$ (Student's *t* test).

© The Author(s)

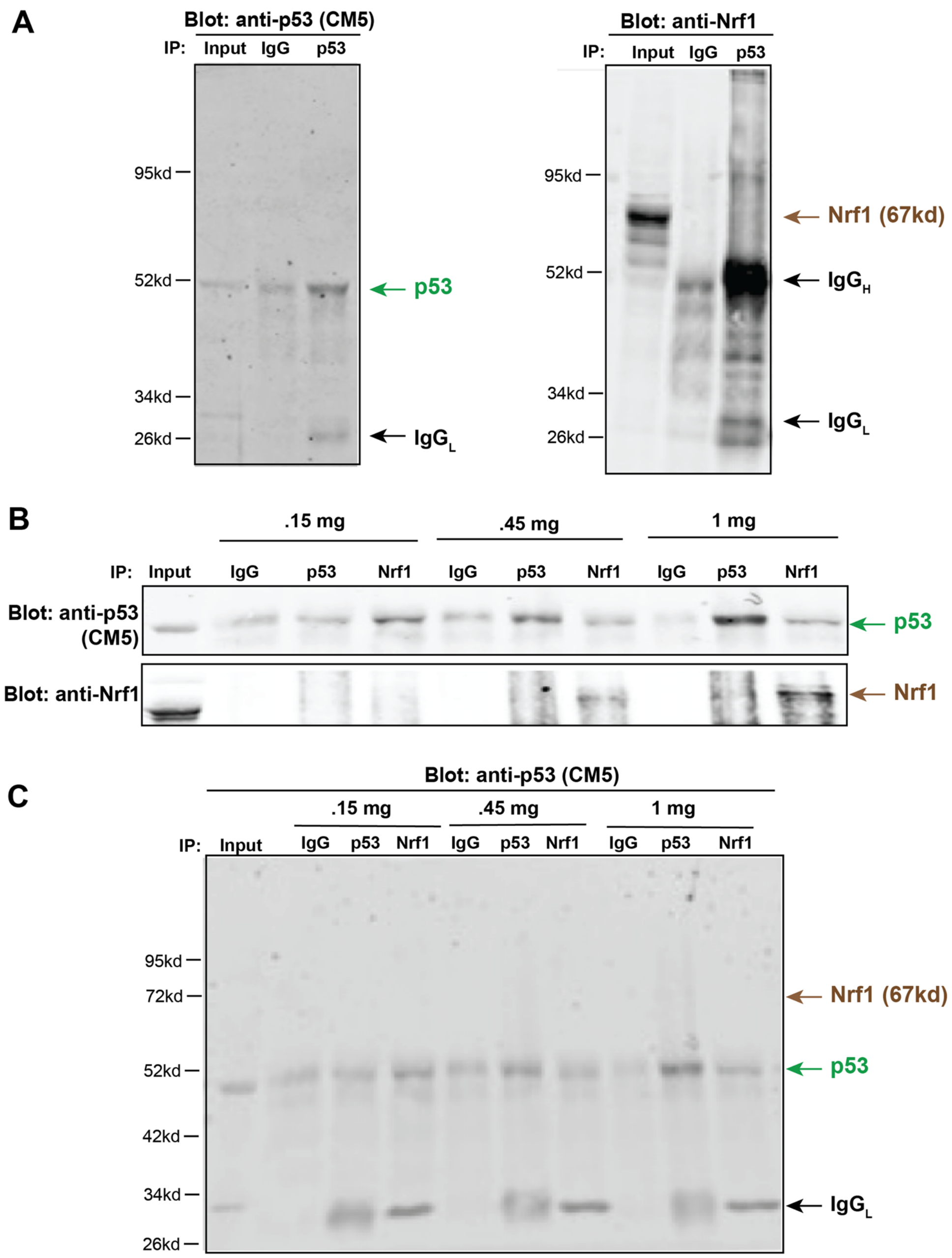

Figure EV3. The antibody to p53 used for chromatin immunoprecipitation does not cross-react with Nrf1.

(**A**) Protein extracts were prepared from the mouse metastatic esophageal squamous carcinoma cell line 378 and subjected to immunoprecipitation with CM5, an antibody to mouse p53 that was used in the chromatin immunoprecipitation assays or an IgG negative control. 10% of the extract that is used for immunoprecipitation is shown as Input. Immunoprecipitates were then subject to immunoblotting with either the antibody to p53 (CM5, on the left) or to Nrf1 (on the right). (**B**) The indicated amounts of protein extracts of 378 cells were subjected to immunoprecipitation with the shown antibodies. Immunoprecipitates were subjected to immunoblotting with either anti-p53 (CM5) or anti-Nrf1 antibodies as shown. (**C**) The immunoblot in (**B**) that was probed with the antibody to p53 prior to cropping is shown. The size of mouse Nrf1 (67kd) is indicated on the right. The position of standard molecular weight markers is shown on the left.

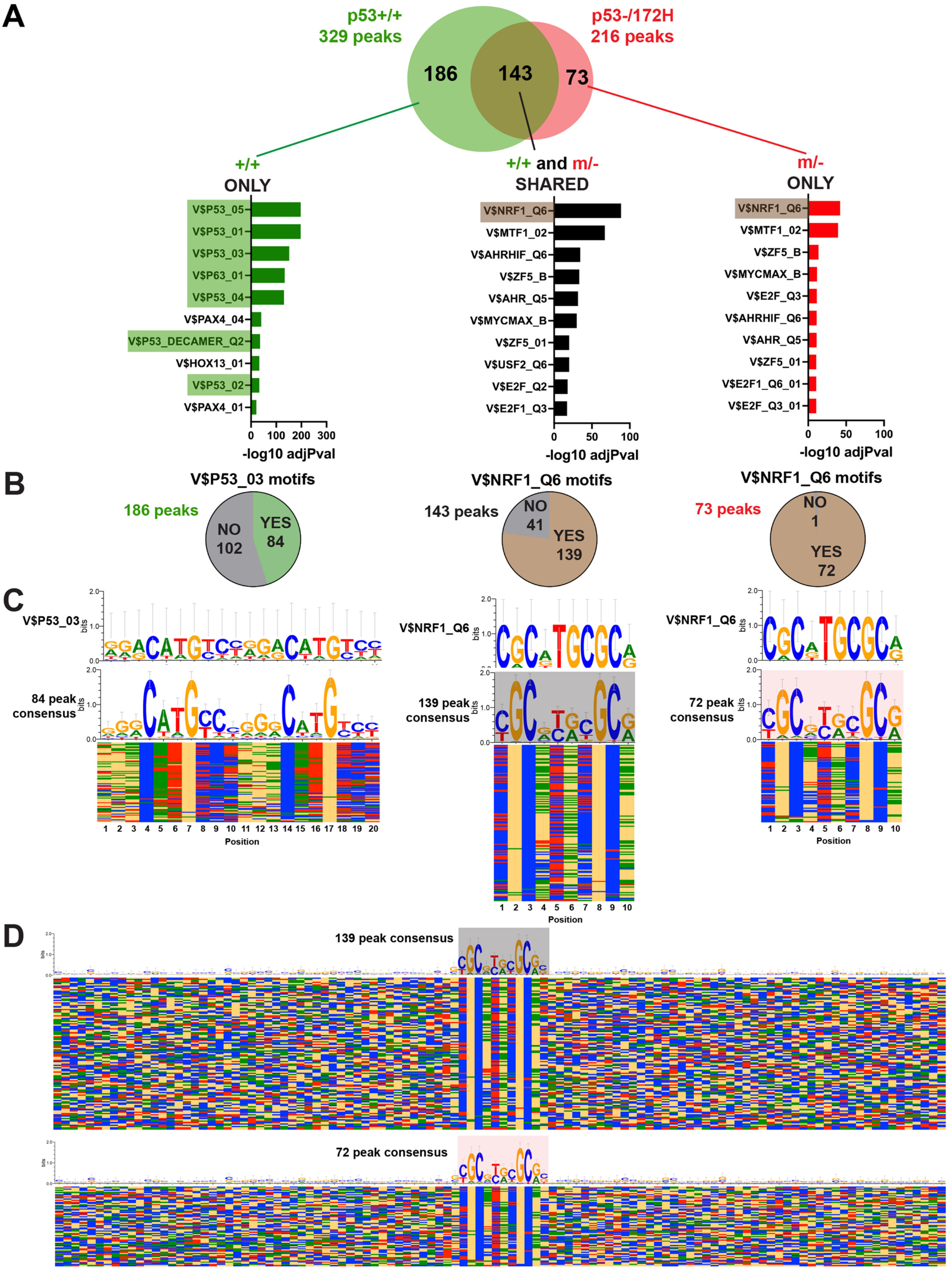
A
p53+/+
329 peaks
p53-/172H
216 peaks
186
143
73
+/+
ONLY
+/+ and m/-
SHARED
m/-
ONLY
V$P53_05
V$P53_01
V$P53_03
V$P63_01
V$P53_04
V$PAX4_04
V$P53_DECAMER_Q2
V$HOX13_01
V$P53_02
V$PAX4_01
0 100 200 300
-log10 adjPval
V$NRF1_Q6
V$MTF1_02
V$AHRHIF_Q6
V$ZF5_B
V$AHR_Q5
V$MYCMAX_B
V$ZF5_01
V$USF2_Q6
V$E2F_Q2
V$E2F1_Q3
0 50 100
-log10 adjPval
V$NRF1_Q6
V$MTF1_02
V$ZF5_B
V$MYCMAX_B
V$E2F_Q3
V$AHRHIF_Q6
V$AHR_Q5
V$ZF5_01
V$E2F1_Q6_01
V$E2F_Q3_01
0 50 100
-log10 adjPval
B
V$P53_03 motifs
186 peaks
NO 102
YES 84
V$NRF1_Q6 motifs
143 peaks
NO 41
YES 139
V$NRF1_Q6 motifs
73 peaks
NO 1
YES 72
C
V$P53_03
84 peak consensus
Position
V$NRF1_Q6
139 peak consensus
Position
V$NRF1_Q6
72 peak consensus
Position
D
139 peak consensus
72 peak consensus

 © The Author(s)

Figure EV4. Wild-type and mutant p53 show overlapping gene occupancies involving sequences that match an Nrf1 consensus motif.

(**A**) 8 week-old mice of the indicated genotype were treated with 9.5 Gy of X-ray. After 3 h, bone marrows were subjected to cross-linking and ChIP-seq analyses. A Venn diagram is shown for genotype-specific and overlapping ChIP-seq peaks with an adjusted *P* value <0.2. The genomic sequences underneath the peaks detected by ChIP-seq were subjected to TRAP analysis. The top matrices that were detected with the corresponding adjPvalues are shown. Statistical analysis for motif analysis was done by TRAP (Transcription Factor Affinity Prediction) using web tools provided by the Max Planck Institute for Molecular Genetics (http://trap.molgen.mpg.de) (Thomas-Chollier et al, 2011). (**B**) As determined by TRAP analysis, the number of peaks which contain a consensus p53 motif (V$P53_03) or a consensus Nrf1 (V&NRF1_Q6) are shown as pie charts. (**C**) The sequences for the peaks with either a canonical p53 or Nrf1 motif, as indicated, were subjected to WebLogo3 analysis and the resulting consensus is shown. For comparison, the TRANSFAC consensus p53 motif (V$P53_03) or consensus Nrf1 (V&NRF1_Q6) are shown. Heat maps for the actual sequences are presented below the consensus. (**D**) An additional 50 bp surrounding the sequences for the peaks with Nrf1 motif were subjected to WebLogo3 analysis and the resulting consensus is shown. Heat maps for the actual sequences are presented below the consensus.

© The Author(s)

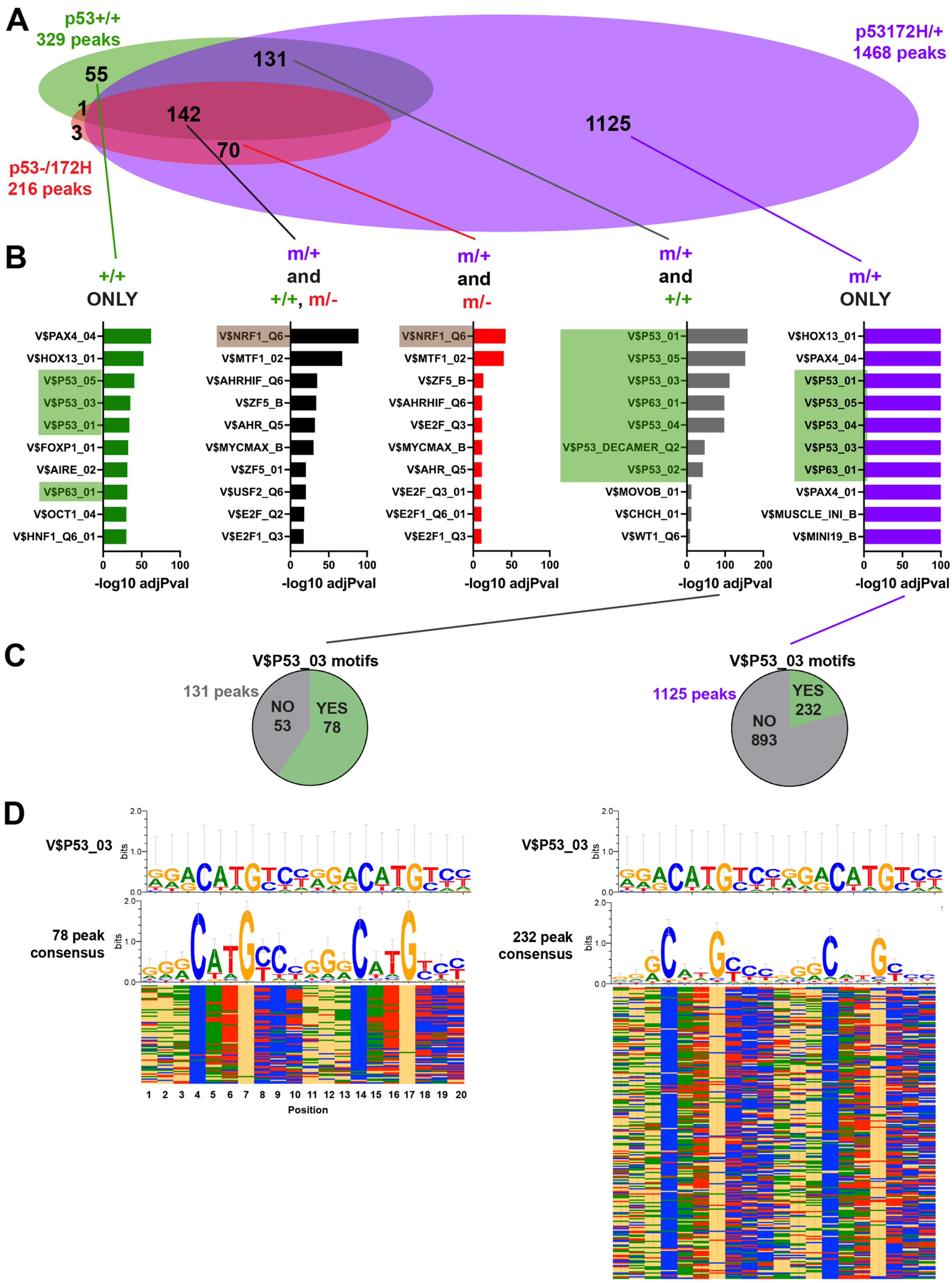
A
p53+/+
329 peaks
p53172H/+
1468 peaks
p53-/172H
216 peaks
55
131
1
3
142
70
1125
B
+/+
ONLY
m/+
and
+/+, m/-
m/+
and
m/-
m/+
and
+/+
m/+
ONLY
V$PAX4_04
V$HOX13_01
V$P53_05
V$P53_03
V$P53_01
V$FOXP1_01
V$AIRE_02
V$P63_01
V$OCT1_04
V$HNF1_Q6_01
V$NRF1_Q6
V$MTF1_02
V$AHRHIF_Q6
V$ZF5_B
V$AHR_Q5
V$MYCMAX_B
V$ZF5_01
V$USF2_Q6
V$E2F_Q2
V$E2F1_Q3
V$NRF1_Q6
V$MTF1_02
V$ZF5_B
V$AHRHIF_Q6
V$E2F_Q3
V$MYCMAX_B
V$AHR_Q5
V$E2F_Q3_01
V$E2F1_Q6_01
V$E2F1_Q3
V$P53_01
V$P53_05
V$P53_03
V$P63_01
V$P53_04
V$P53_DECAMER_Q2
V$P53_02
V$MOVOB_01
V$CHCH_01
V$WT1_Q6
V$HOX13_01
V$PAX4_04
V$P53_01
V$P53_05
V$P53_04
V$P53_03
V$P63_01
V$PAX4_01
V$MUSCLE_INI_B
V$MINI19_B
-log10 adjPval
C
V$P53_03 motifs
131 peaks
NO 53
YES 78
V$P53_03 motifs
1125 peaks
YES 232
NO 893
D
V$P53_03
78 peak consensus
V$P53_03
232 peak consensus
bits
Position
1 2 3 4 5 6 7 8 9 10 11 12 13 14 15 16 17 18 19 20

 © The Author(s)

Figure EV5. In the presence of wild-type p53, mutant p53 shows increased gene occupancies involving sequences that match a p53 consensus motif.

(**A**) 8 week-old mice of the indicated genotype were treated with 9.5 Gy of X-ray. After 3 h, bone marrows were subjected to cross-linking and ChIP-seq analyses. A Venn diagram is shown for genotype-specific and overlapping ChIP-seq peaks with an adjusted *P* value <0.2. Statistical analysis for motif analysis was done by TRAP (Transcription Factor Affinity Prediction) using web tools provided by the Max Planck Institute for Molecular Genetics (http://trap.molgen.mpg.de) (Thomas-Chollier et al, 2011). (**B**) The genomic sequences underneath the peaks detected by ChIP-seq were subjected to TRAP analysis. The top matrices that were detected with the corresponding adjPvalues are shown. (**C**) As determined by TRAP analysis, the number of peaks which contain a consensus p53 motif (V$P53_03) are shown as pie charts. (**D**) The sequences for the peaks with a canonical p53 were subjected to WebLogo3 analysis and the resulting consensus is shown. For comparison, the TRANSFAC consensus p53 motif (V$P53_03) is shown. Heat maps for the actual sequences are presented below the consensus.

© The Author(s)
